# Optimal Specimens and Lesions for Mpox Diagnosis Using Real-Time PCR, South Korea

**DOI:** 10.3201/eid3203.250582

**Published:** 2026-03

**Authors:** Dong-Min Kim, Munawir Muhammad, Jin Won Kim, Choon-Mee Kim, Jun-Won Seo, Da Young Kim, Na Ra Yun, Beomgi Lee, Minji Lee, Jeong Hyun Lee, Myung-Min Choi, Yoon-Seok Chung

**Affiliations:** Chosun University College of Medicine, Gwang-Ju, South Korea (D.-M. Kim, M. Muhammad, C.-M. Kim, J.-W. Seo, D.Y. Kim, N.R. Yun, B. Lee); Hasanuddin University, Makassar, Indonesia (M. Muhammad); Korea Disease Control and Prevention Agency, Cheongju, South Korea (J.W. Kim, M. Lee, J.H. Lee, M.-M. Choi, Y.-S. Chung)

**Keywords:** Mpox, monkeypox virus, viruses, specimens, Ct value, real-time PCR, South Korea

## Abstract

We analyzed 612 specimens from 135 patients with monkeypox virus clade IIb in South Korea by using real-time PCR. Crusted and anogenital skin lesions and rectal swab specimens demonstrated the highest positivity rates. Viral loads varied by lesion type, anatomic site, and time since symptom onset, supporting our specimen selection for clade IIb detection.

Mpox, a zoonotic disease caused by the monkeypox virus (MPXV), a member of the genus *Orthopoxvirus*, was first reported in South Korea in 2022 and initially involved imported cases and a hospital-acquired infection (*1**,**2*). A local outbreak began in April 2022 and peaked in late April to early May 2023, largely affecting young adult men (mostly men who have sex with men); most transmission events were linked to sexual contact (*2*,*3*,*4*). Genomic analysis revealed that circulating strains in 2023 belonged to MPXV clade IIb, with the B.1.3 lineage predominating (*4*).

As the outbreak evolved, understanding the MPXV transmission dynamics and diagnostic accuracy became increasingly crucial. Although mpox is primarily transmitted by symptomatic persons, presymptomatic transmission occurring 1–4 days before symptom onset has been reported (*5*). Accurate diagnosis relies on appropriate specimen selection and timing of sample collection. However, relatively few studies have systematically compared viral loads across specimen types, and we did not find published studies that comprehensively evaluated differences between anogenital and nonanogenital skin lesions. To address those gaps, we assessed MPXV viral loads and positivity rates in South Korea by specimen type and time since symptom onset by using real-time quantitative PCR (qPCR).

## The Study

We conducted a retrospective observational study to analyze demographic characteristics, symptom onset, specimen types, qPCR results, and cycle threshold (Ct) values of suspected MPXV cases in South Korea referred to the Korea Disease Control and Prevention Agency (KDCA). Clinical specimens were collected by trained healthcare personnel and included skin lesions, upper respiratory tract swab specimens, blood, and rectal swab specimens. We categorized skin lesions by type (crusted, vesicular, papular, pustular, or unknown) and site (anogenital, nonanogenital, or unknown), including the anus, perineum, penis, and vulva. We grouped samples by days since symptom onset (0–1, 2–3, 4–10, and >10 days). We considered Ct values >40 or undetectable negative and Ct values <40 positive.

We extracted viral DNA from 200 µL of clinical specimens by using the QIAamp Blood Mini Kit (QIAGEN, https://www.qiagen.com) following the manufacturer’s protocol. qPCR targeted F3L and A39R genes by using the following primers and probes: F3L forward 5′-CATCTATTATAGCATCAGCATCAGA-3′, reverse 5′-GATACTCCTCCTCGTTGGTCTAC-3′, probe 5′-FAM-TGTAGGCCGTGTATCAGCATCCATT-IBFQ-3′; A39R forward 5′-TGGGATAACGAATCCAATGTCA-3′, reverse 5′-GCGTGCTTCCAGCAACACT-3′, probe 5′-FAM-AGCGCCTAGCACAGAACACATTTACGA-IBFQ-3′.

For group comparisons, we used χ^2^ or Fisher exact test for categorical variables and the Kruskal-Wallis test or analysis of variance for Ct value comparisons. We conducted analyses by using SPSS Statistics 29.0.10 (IBM, https://www.ibm.com) and GraphPad Prism version 8 (https://www.graphpad.com). This study was approved by the KDCA Institutional Review board (approval no. 2020-03-01-P-A), which waived written informed consent.

During August 28, 2022–October 3, 2023, the KDCA received 1,668 specimens from 379 patients with suspected mpox. After we excluded 244 patients (1,056 specimens) because of missing clinical data (999 samples from 231 patients) and unclear onset times (57 samples from 13 patients), we analyzed 612 specimens from 135 patients. Most (97.7%) patients were men. Median patient age was 31.7 (interquartile range [IQR] 27.7–37.1) years, and median collection time was 6 (IQR 2.0–8.0) days after onset. Specimens were 362 skin lesions, 109 upper respiratory tract swab specimens, 135 blood samples, and 6 rectal swab specimens; 445 samples were PCR-positive.

**Figure 1:**
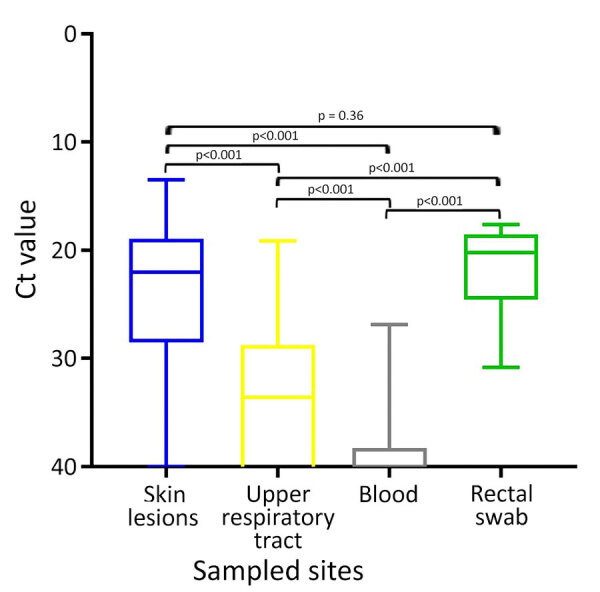
Comparison of Ct values of real-time PCR by lesion and time point after symptom onset in patients with mpox, South Korea, 2022–2023. Horizontal lines within boxes represent medians, box tops and bottoms represent interquartile ranges, and error bars represent minimum and maximum values. Ct, cycle threshold.

MPXV DNA was more frequently detected from skin lesions (92.8%, n = 336/362) and rectal swab specimens (100%, n = 6/6) than from upper respiratory tract (68.8%, n = 75/109) and blood samples (28.2%, n = 38/135) (Table 1; Appendix Figure 1). Median Ct values were significantly lower for skin lesions (median 22.01, IQR 19.25–28.19) and rectal swab specimens (median 20.19, IQR 18.85–24.23) than in the upper respiratory tract (median 33.61, IQR 29.09–40.00) and blood samples (median 40.00, IQR 38.61–40.00; p<0.001); we found no significant difference between skin lesions and rectal swab specimens (p = 0.36) (Table 1; Figure 1). Among skin lesions, crusted types demonstrated the highest positivity rate (93.3%, n = 70/75) and the lowest median Ct value (median 20.63, IQR 18.30–24.48), significantly lower than for other lesion types (median 23.33, IQR 19.74–30.00; p<0.001). Anogenital lesions had lower Ct values (median 20.78, IQR 17.91–25.65) than lesions at other sites (median 22.82, IQR 19.91–29.94; p = 0.018). Positivity rates (p = 0.556) and Ct values (p = 0.351) did not differ significantly between oropharyngeal and nasopharyngeal specimens; however, oropharyngeal samples tended toward higher positivity (64.3%) and lower Ct values (median 34.37, IQR 29.78–40.00) compared with nasopharyngeal samples (50.0%, n = 2/4; median Ct 37.14, IQR 32.45–40.00), despite the limited sample size.

**Table 1:** Mpox specimen types and Ct values of 135 confirmed patient samples acquired from the Korea Disease Control and Prevention Agency when a suspected mpox patient visited the hospital, South Korea, 2022–2023*

Specimen type	Samples		χ^2^	Ct values					Median (IQR) days after symptom onset
	Total	No. (%) positive		Mean (SD)	Median (95% CI)	25th percentile	75th percentile	p value	
All skin lesions	362	336 (92.82)		24.41 (6.90)	22.01 (21.55–22.87)	19.25	28.19		6 (2.00–8.00)
Type									
Crust	75	70 (93.33)	0.2965	22.51 (6.52)	20.63 (19.59–21.76)	18.30	24.48	<0.0001	7.00 (2.00–13.00)
Other	260	239 (91.92)		25.32 (7.02)	23.33 (22.11–24.6)	19.74	30		5.00 (2.00–7.00)
Unknown	27	27 (100)		20.9 (4.20)	19.85 (19.17–21.74)	18.87	21.85		7.00 (5.00–10.00)
Site									
Anogenital	74	72 (97.30)	0.1142	22.58 (6.15)	20.78 (19.71–22.06)	17.91	25.65	0.0178	6.00 (3.00–7.25)
Other	116	109 (93.97)		25.14 (6.91)	22.82 (21.51–24.91)	19.91	29.94		5.00 (2.25–7.75)
Unknown	172	155 (90.12)		24.7 (7.10)	22.51 (21.16–24.25)	19.47	28.71		6.00 (1.00–8.00)
Nationality									
South Korea	338	314 (92.90)	0.8154	24.24 (6.84)	21.87 (21.66–22.77)	19.24	27.7	0.2068	
Other Asian	21	19 (90.50)		26.57 (7.47)	25 (20.85–30.25)	20.75	30.46		
Western countries	3	3 (100)		28.32 (8.42)	30.39 (19.06–35.52)	19.06	35.52		
Sample location									
Upper respiratory tract	109	75 (68.81)	0.5564	33.29 (6.05)	33.61 (32.52–35.24)	29.09	40	0.3505	5.00 (2.00–8.00)
Nasopharynx	4	2 (50)		36.53 (4.13)	37.14 (31.84–40.00)	32.45	40		5.00 (0.75–7.75)
Oropharynx	28	18 (64.29)		34.35 (5.11)	34.37 (31.22–40.00)	29.78	40		3.00 (0.00–8.00)
Unknown	77	55 (71.43)		32.74 (6.39)	33.16 (31.44–35.24)	27.56	40		6.00 (3.00–8.00)
Blood	135	38 (28.19)	NA	38.75 (2.56)	40.00 (40.00–40.00)	38.61	40.00	NA	5.00 (3.00–7.00)
Rectal swab	6	6 (100)	NA	21.68 (4.708)	20.19 (17.61–30.82)	18.85	24.23	NA	5.00 (1.50–12.25)

*Ct, cycle threshold; IQR, interquartile range; NA, not available.

**Figure 2:**
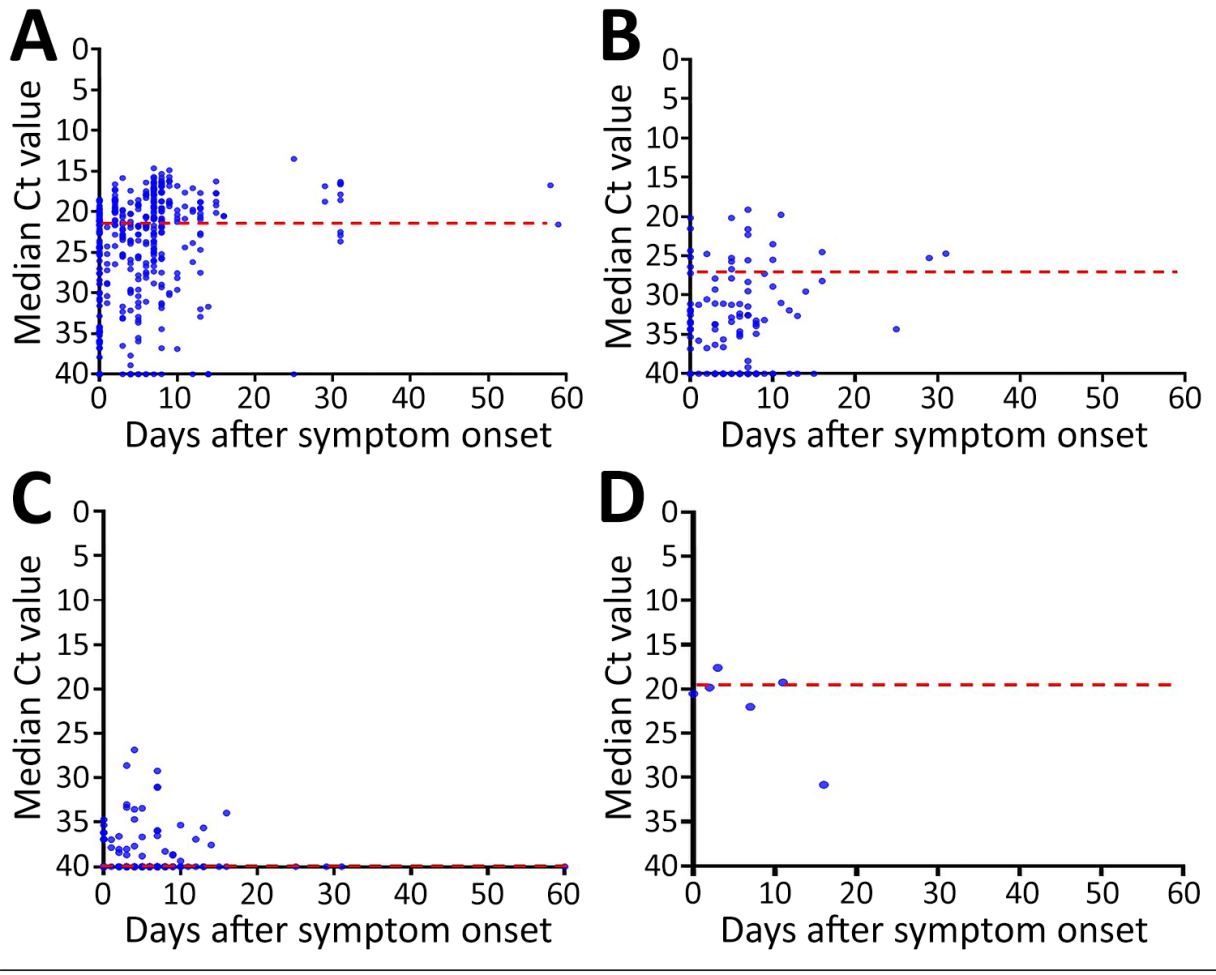
Monkeypox viral loads given as Ct values in patients with mpox, according to sampled sites and time of onset, South Korea, 2022–2023. Blue dots indicate Ct results for each sample. Red dashed lines represent median values. Ct, cycle threshold.

Positivity and Ct values varied over time after symptom onset (Table 2; Figure 2). The Ct values of the skin lesions changed significantly (p<0.001), whereas positivity remained high (≈90%) for up to 60 days (p = 0.142). Upper respiratory tract specimens showed no significant changes in positivity (p = 0.738) or Ct values (p = 0.369), with peak positivity (75%) on day 1, lasting until day 30. Blood samples had low positivity (28.2%) and no substantial variation over time, peaking in the first 3 days (32%), and were detectable for ≈20 days.

**Table 2:** Comparison of the positivity rate and Ct value of real-time PCR at the time point of each specimen after symptom onset in patients with mpox, South Korea, 2022–2023*

Specimen type	Days since symptom onset											χ^2^	p value
	0–1			2–3			4–10			10			
	No. (%) positive	Median (IQR) Ct		No. (%) positive	Median (IQR) Ct		No. (%) positive	Median (IQR) Ct		No. (%) positive	Median (IQR) Ct		
Skin lesion, n = 362	67/76 (88.2)	27.38 (21.9–34.8)		47/48 (97.9)	21.41 (19.5–25.0)		177/188 (94.2)	21.7 (18.8–27.4)		45/50 (90.0)	20.18 (17.8–24.0)	0.1421	<0.0001
Upper respiratory tract, n = 109	18/24 (75.0)	33.47 (28.2–39.2)		10/15 (66.7)	34.34 (30.5–40.0)		37/57 (64.9)	33.9 (29.2–40.0)		10/13 (76.9)	30.98 (25.0–37.2)	0.7380	0.3689
Blood, n = 135	8/25 (32.0)	40 (36.9–40)		8/22 (36.4)	40 (38.0–40)		18/74 (24.3)	40 (39.84–40)		4/14 (28.6)	40 (36.6–40)	0.6930	0.7635
Rectal swab, n = 6	1/1 (100)	20.54 (20.5–20.5)		2/2 (100)	18.7 (17.6–19.8)		1/1 (100)	22.0 (22.0–22.0)		2/2 (100)	26.43 (22.0–30.8)	NA	0.1778

*Denominators indicate no. specimens tested on those days. χ^2^ value is of positivity rate; p value is of Ct. Ct, cycle threshold; IQR, interquartile range; NA, not available.

Mpox is primarily transmitted through direct contact with body fluids, respiratory droplets, or contaminated surfaces (*6*). Increasing evidence indicates that sexual contact is the dominant transmission route during outbreaks, particularly among men who have sex with men (*7*). This evidence is supported by frequent anogenital lesions and the detection of culturable viruses in saliva, the nasopharynx, and semen (*7*). The high positivity and low Ct values observed in anogenital lesions in our study further support those findings.

After inoculation, MPXV replicates in the skin, spreads to the lymph nodes, and causes viremia and systemic dissemination (*8*). Skin lesions progress through macules, papules, vesicles or pustules, and crusts (*6*). Crusted lesions have historically been regarded as less infectious, but accumulating evidence indicates that viral DNA and infectious virus might persist until crust detachment (*9*,*10*). Consistent with that persistence of infectious virus, we observed high MPXV DNA levels in crusted lesions, indicating its potential infectivity. MPXV also demonstrates strong tropism for keratinocytes. Skin specimens, particularly anogenital lesions and rectal swab specimens, exhibit higher viral loads than blood or respiratory specimens, underscoring the central role of the skin in viral replication and direct contact transmission (*11*,*12*).

Ct values in skin lesions decreased significantly over time, consistent with reports from China demonstrating high viral DNA detection for up to 3 weeks (*13*). Upper respiratory tract samples peaked in positivity 1 day after symptom onset and then declined, similar to the oropharyngeal patterns reported in China (*13*). Blood samples demonstrated low positivity, which increased slightly in the first 3 days and then declined, consistent with reports of plasma and serum viral DNA peaking before day 10 (*13*). Our findings support evidence that skin and rectal swab specimens yield higher viral loads than blood or respiratory swab specimens (*14*,*15*).

The limitations of our study include incomplete clinical data and a small number of rectal swabs. For many patients, we relied on referral records that lacked detailed lesion or location data. Prospective studies are necessary to clarify the links between symptoms, severity, duration, and outcomes.

## Conclusions

Our study of MPXV clade IIb infection during the 2022–2023 outbreak in South Korea found that crusted and anogenital skin lesions and rectal swab specimens demonstrated the highest PCR positivity rates, followed by upper respiratory tract and blood samples. Peak positivity occurred around day 1 in respiratory specimens and on day 3 in blood. The Ct values differed significantly by lesion type and anatomic location, highlighting the importance of targeted specimen collection and the appropriate sampling timing in MPXV clade IIb infection.

Appendix Additional information about optimal specimens and lesions for mpox diagnosis using real-time PCR, South Korea.
