# Supplementary material for: Optimal Specimens and Lesions for Mpox Diagnosis Using Real-Time PCR, South Korea
Source: Emerg Infect Dis. 2026 Mar;32(3):404–8. doi: 10.3201/eid3203.250582 (PMC13016027; doi:10.3201/eid3203.250582)
Supplement: Appendix — Additional information about optimal specimens and lesions for mpox diagnosis using real-time PCR, South Korea. [file 25-0582-Techapp-s1.pdf]

Article DOI: https://doi.org/10.3201/eid3203.250582
*EID cannot ensure accessibility for supplementary materials supplied by authors. Readers who have difficulty accessing supplementary content should contact the authors for assistance.*

# Optimal Specimens and Lesions for Mpox Diagnosis Using Real-Time PCR, South Korea

## Appendix

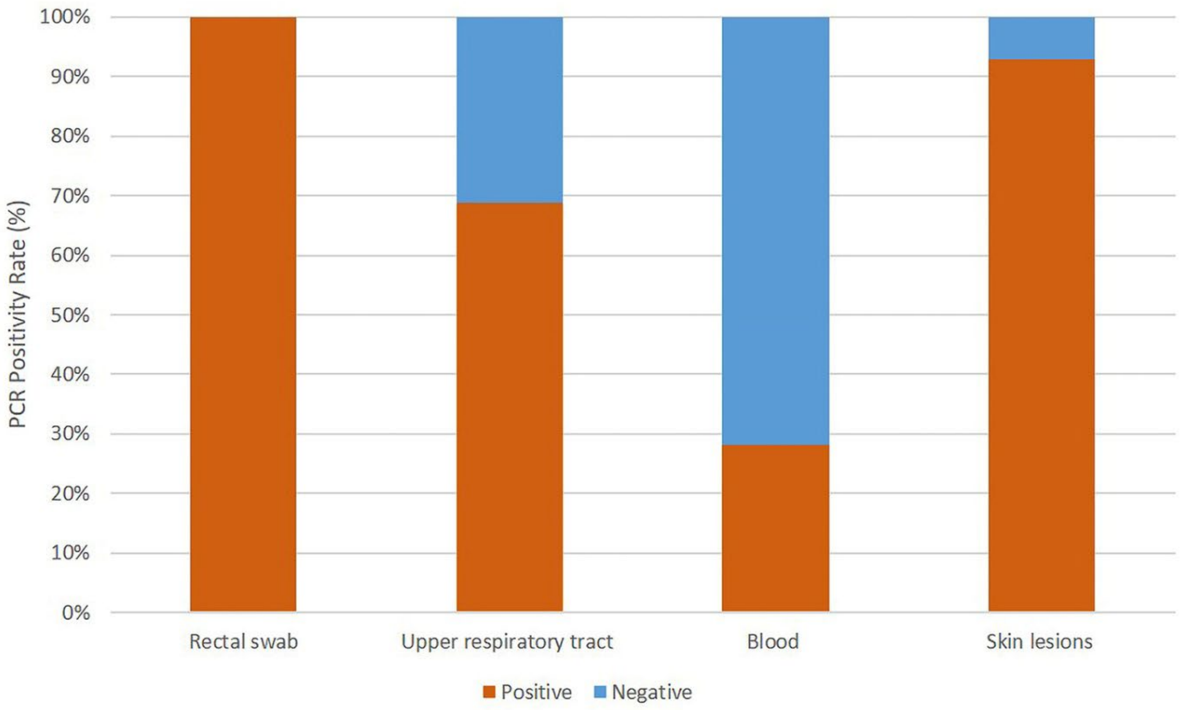


**Appendix Figure.** Monkeypox virus PCR positivity rate by sampling site.
